# Validation of an MRI-based classification of peroneus brevis tendon morphology: a four-type system with high inter-rater reliability

**DOI:** 10.1007/s00256-025-05010-4

**Published:** 2025-08-13

**Authors:** Rafał Zych, Dan Mocanu, Ymer Hagberg, Katarzyna Bokwa-Dąbrowska, Michael Huuskonen, Isaac Romanus, Dawid Dziedzic, Pawel Szaro

**Affiliations:** 1 Department of Clinical and Descriptive Anatomy, Medical University of Warsaw, Warsaw, Poland; 2 Department of Musculoskeletal Radiology, Sahlgrenska University Hospital, Gothenburg, Sweden; 3 Department of Radiology, Institute of Clinical Sciences, Sahlgrenska Academy, University of Gothenburg, Gothenburg, Sweden

**Keywords:** MRI-based classification, Peroneus brevis tendon, Tendon morphology, Sports imaging, Inter-rater agreement, Magnetic resonance imaging

## Abstract

**Objectives:**

Split tears of the peroneus brevis tendon are a common and often underrecognized cause of lateral ankle pain and instability in athletes. Although MRI is widely used for ankle assessment, no validated system exists for classifying normal morphological variation of the peroneus brevis tendon. This study aimed to validate an MRI-based classification system.

**Methods:**

We analyzed 130 normal peroneus brevis tendons (power > 0.8, *α* = 0.05, effect size = 0.31) on consecutive 3T ankle MRI scans. Persons with fractures, ligament injuries, or peroneal tendon pathology were excluded. Each tendon was evaluated on a single standardized axial PD-weighted slice, selected just proximal to the lateral malleolus apex. Seven independent raters classified tendons into four types: general flat, flat with lateral bulge, flat with medial bulge, and oval. Inter-rater reliability was assessed using Cohen’s kappa, Gwet’s AC1, and Fleiss’ kappa. Classification robustness was evaluated using F1 scores, ROC analysis, and precision-recall metrics.

**Results:**

The most common forms were general flat (37.7%) and oval (22.3%). Fleiss’ kappa was 0.691 (95% CI 0.629–0.752), with strong pairwise agreement (Cohen’s kappa and Gwet’s AC1: 0.58–0.87). ROC AUC was 0.89; majority voting improved agreement (F1 = 0.93).

**Conclusions:**

This classification system is robust and reliable, supporting consistent MRI-based assessment of tendon morphology in clinical and research settings.

**Supplementary Information:**

The online version contains supplementary material available at 10.1007/s00256-025-05010-4.

## Introduction

Peroneus brevis split tears are a common but frequently underdiagnosed cause of chronic lateral ankle pain and instability in athletes [1–4]. These injuries often arise after ankle sprains and may persist for years if not properly recognized, compromising performance and delaying return to sport [5]. Variations in tendon morphology, particularly flattening, may predispose to split tears by increasing mechanical stress within the retromalleolar groove [6]. Despite this, no validated classification of peroneus brevis tendon shape exists at the level where split tears typically originate [7]. A reliable, reproducible classification system may support research on peroneal tendon morphology and pathology.

Previous studies have used terms such as “boomerang,” “chevron,” or “bifid” to describe the shape of the peroneus brevis tendon on axial MRI [7, 8]. While these descriptors suggest that cross-sectional shape may be relevant in identifying pathology, the terminology is inconsistent and not standardized. In some cases, such as the “boomerang” shape, there may already be a split tear, whereas in others the distinction between variant anatomy and disease is unclear. To date, no validated classification system has been proposed to describe normal morphological variation of the peroneus brevis tendon, which limits diagnostic consistency and research comparability.

Despite the clinical significance, peroneus brevis split tear remains challenging due to the complex anatomical relationship of the peroneal tendons and the inherent limitations of the available imaging modalities [9]. The peroneus brevis and peroneus longus tendons lie adjacent to each other, tightly confined within the retromalleolar groove and stabilized posteriorly by the superior peroneal retinaculum [10, 11]. Tight anatomical relationships between the content of the superior peroneal tunnel make it difficult to assess peroneus brevis pathology on ultrasound and MRI [12].

MRI and ultrasound are both commonly used to assess peroneal tendon tears, but comparisons across studies are limited by differences in technique, equipment, and reader expertise. MRI sensitivity for peroneus brevis tears ranges from 57 to 100% [13, 14], while ultrasound shows 88–100% sensitivity in smaller, expert-performed studies [14–17]*.* In a recent study with surgical correlation, consensus MRI interpretation achieved 84% sensitivity and 92% specificity for peroneus brevis tears, but only 36% sensitivity for peroneus longus tears [12]. Diagnostic accuracy varied across radiologists, highlighting interobserver variability and the potential value of consensus reading. Both modalities have technical challenges: MRI is affected by the magic angle phenomenon, which can obscure pathology, while ultrasound is susceptible to anisotropy, requiring precise probe orientation.

According to recommendations, the term “tear” refers to a partial or longitudinal tear, while “rupture” is generally used to describe a complete discontinuity of the tendon, with full separation of its ends [18, 19]. Non-complete tears are particularly difficult to detect with imaging, as their radiological features may overlap with normal anatomical variations. One proposed imaging marker is an alteration in tendon shape on transverse cross section on MRI [7]. Despite clinical observations, there is no consensus in the literature regarding the normal anatomical variability of the peroneus brevis tendon. Its shape has been hypothesized to influence the susceptibility to split tears, with some authors suggesting that flatter peroneus brevis tendons are at greater risk due to compression by the peroneus longus against the retromalleolar sulcus [20]. However, there is no validated classification system for peroneus brevis morphology, nor any systematic studies evaluating whether anatomical variants contribute to the risk of split tear formation.

The morphology of the retromalleolar groove, a low-lying muscle belly, and the presence of a peroneus quartus muscle have been frequently discussed in the literature [11, 19, 21–24]. Although a shallow sulcus or sharp groove contour has been associated with peroneal tendon instability in some studies, these anatomical factors remain controversial, with inconsistent findings across publications. While several anatomical variations have been explored, the specific variability of the normal peroneus brevis tendon itself has received comparatively little attention.

The lack of a standardized classification system for the peroneus brevis tendon morphology represents a significant gap in musculoskeletal radiology. Given the importance of reliable and reproducible imaging criteria, any proposed classification must undergo rigorous validation to ensure its applicability in both clinical and research settings. To our knowledge, no studies have systematically classified peroneus brevis tendon morphology on MRI. Thus, we addressed this gap by proposing a classification system of peroneus brevis tendon shape variability and evaluating its potential clinical implications. We aimed to assess the reliability of our proposed peroneus brevis tendon classification system, to identify potential misclassification patterns, and to explore factors that may influence classification accuracy.

## Methods

### Ethical considerations

All procedures performed in studies involving human participants were in accordance with the ethical standards of the institutional and/or national research committee and with the 1964 Helsinki Declaration and its later amendments or comparable ethical standards. The National Ethical Authority approved this study and waived informed consent (Dnr 2024–07283-02).

### Study design and observer validation framework

This study evaluated the inter-rater agreement regarding a proposed classification of the peroneus brevis tendon form on the transverse section at the level of the lateral malleolus. This study was conducted and reported in accordance with the Guidelines for Reporting Reliability and Agreement Studies (GRRAS) [25].

### MRI examinations

The dataset was compiled retrospectively from MRI examinations performed at our institution in 2021–2024 with a 3 T machine (Philips Ingenia) due to sports injuries or pain related to physical activity. MRI examinations were performed using a dedicated ankle coil, which ensures consistent positioning of the ankle joint. Wedge-shaped cushions placed inside the coil prevent movement or changes in the ankle during the examination. Axial proton density–weighted images were acquired with a turbo spin echo sequence using an echo time of 45 ms and a repetition time of 2800–5000 ms. The voxel dimensions were 0.45 × 0.53 × 3.0 mm, with a slice thickness of 3 mm and a field of view of 14 cm. No interslice gap was used in the protocol. We retrospectively reviewed 439 ankle MRI examinations performed at our institution between January 2021 and December 2024. All examinations followed our standard ankle MRI protocol and had been conducted due to trauma or pain in the ankle. Only one MRI per patient was included. Exclusions were examinations showing abnormal T2 or proton density signal, tenosynovitis, or tendinosis—were excluded. Additional exclusion criteria included the presence of fractures, infections, tumors, transverse tendon ruptures, postoperative changes, metal or motion artifacts, duplicate scans from the same patient, age under 18 years, or missing clinical information (Flowchart in Supplementary material Figure S1).

### Raters

Seven independent raters with varying professional backgrounds participated in the classification process. The group included two board-certified musculoskeletal radiologists (Rater 1 with 10 years and Rater 5 with 6 years of experience in musculoskeletal imaging), one radiology resident (Rater 6), one medical doctor (Rater 3), two physiotherapists (Raters 2 and 7), and one fifth-year medical student (Rater 4). All raters underwent a structured training session on the use of the classification system prior to the assessment.

A structured visual assessment approach was used for grading. Each rater independently evaluated the tendons and assigned one of four predefined morphological types. The classification system was developed based on a preliminary study conducted by our team and was further refined through pilot consensus rounds prior to the main study. The reference standard was established through consensus agreement between two experienced musculoskeletal radiologists. To assess inter-rater reliability, each rater independently classified all cases. All other raters performed the classifications independently and were blinded to clinical information, imaging metadata, and each other’s assessments. Intra-rater reliability was assessed after a washout period. To assess intra-rater reliability, a random subset of 30% of cases was re-evaluated by each rater after a three-week washout period [26].

### Data acquisition and preprocessing

#### Sampling

Convenience sampling was used to select all available MRI scans of the ankle that met the inclusion criteria. A power analysis was performed prior to the study to determine the sample size. With power > 0.8, alpha = 0.05, an effect size of 0.31 based on our preliminary study, and a 10% buffer, 130 participants were required.

#### Preprocessing

Analysis of the peroneus brevis tendon shape was based on proton density–weighted axial images. Before starting the main study, we reviewed 40 MRI examinations with normal peroneal tendons to estimate the required effect size. In this preliminary study, we observed that the cross-sectional shape of the tendon remained consistent throughout the short retromalleolar segment. Based on these findings, we considered the use of a single, clearly defined axial slice to be a reliable and reproducible approach for classification across cases. A senior musculoskeletal radiologist selected a representative image at the level of the lateral malleolus, inferior to the syndesmosis, at the level of the ankle joint space.

The selected images were exported, and an electronic form was created, including the images and four classification options (general flat, flat with a medial bulge, flat with a lateral bulge, and oval tendon), which was then distributed to the raters. Each rater received detailed instructions including definitions of tendon forms and examples (not including the evaluated cases) regarding tendon forms and assessed tendons using a standardized protocol for the study.

### Proposed classification

**Fig. 1:**
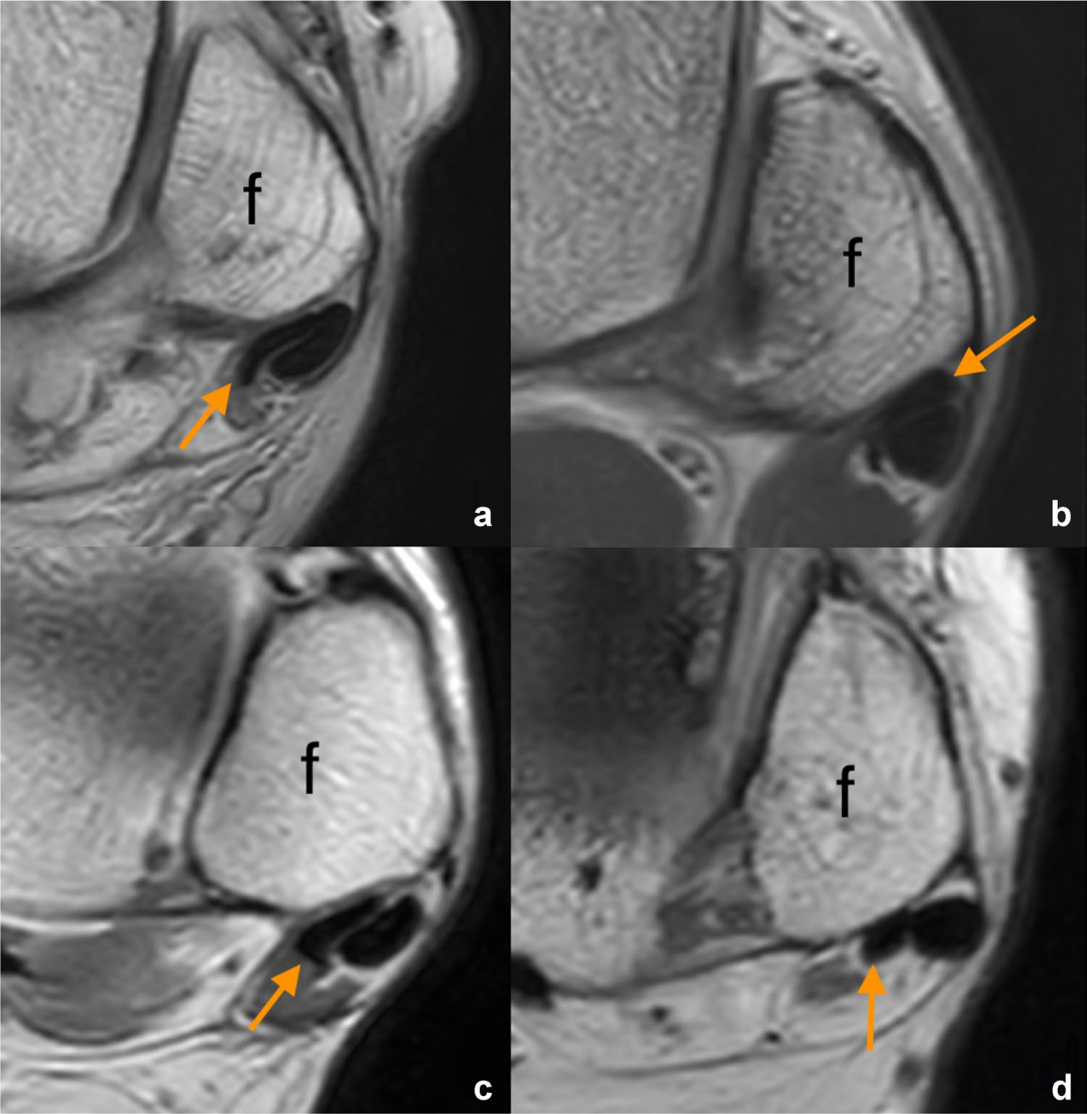
Axial section at the level of the lateral malleolus and the ankle joint space (magnetic resonance images with proton-density weighting, left sides). The four morphological types of the peroneus brevis tendon (arrow) are as follows: **a** general flat, **b** flat with a lateral bulge, **c** flat with a medial bulge, and **d** oval tendon. The fibula is labeled with f

The proposed classification (nominal variable) is based on the visual assessment of the ratio of thickness (the shortest diameter) to the width of the tendon (the longest diameter). The four types are defined below (Fig. 1).

#### General flat tendon

The thickness of the tendon (defined as the anteroposterior [AP] dimension) is relatively consistent across its length. The width of the tendon is significantly greater than its thickness. The tendon in this form can be either straight, curved, or folded.

#### General flat with a lateral bulge

The tendon is flattened, but the lateral edge bulges, increasing the thickness in the lateral part.

#### General flat with a medial bulge

The tendon is flattened, but the medial edge bulges, resulting in an increase in thickness in the medial part.

#### Oval tendon

The peroneus brevis tendon takes the shape of an oval, resembling the cross-section of the peroneus longus tendon. The thickness is slightly smaller than the width.

### Gold standard

The final result was a consensus between two musculoskeletal radiologists, which was the gold standard.

### Generative AI tools

Generative AI tools, specifically ChatGPT (OpenAI, GPT-4 architecture), were used to assist in language correction, improving the clarity and readability of the manuscript. No generative AI tools were used for data analysis, research design, interpretation of results, or the formation of scientific conclusions. The authors carefully reviewed, revised, and approved all AI-generated edits to ensure accuracy and integrity.

### Statistical analysis

R version 4.4.3 (R Core Team, Vienna, Austria) and RStudio version 2024.12.1 + 563 (2024.12.1 + 563) were used for statistical analysis and data visualization. The ggplot2 package was used to visualize the data. Inter-rater agreement across the seven raters was assessed based on Fleiss’ kappa with a bootstrapped 95% confidence interval. For pairwise agreement, Cohen’s kappa and Gwet’s AC1 were calculated between all rater pairs. These statistics were also used to assess intra-rater reliability after a 3-week washout period. Gwet’s AC1 was included as a robust alternative to Cohen’s kappa, particularly considering potential prevalence and marginal distribution imbalances.

The F1 score, defined as the harmonic mean of precision and recall, was calculated for each rater to assess the performance of the proposed classification. This metric reflects the balance between correctly identifying tendon classifications (precision) and detecting all cases of a given class (recall). The F2 score (*β* = 2) was calculated to account for cases where recall was prioritized. In addition, a majority vote approach was applied, where the most frequently assigned tendon form among the seven raters was selected as the predicted class. Then, the majority vote classifications were compared to the gold standard (defined as a consensus between two musculoskeletal radiologists) to calculate the overall classification performance metrics.

Receiver operating characteristic (ROC) curve analysis was conducted by using a cumulative one-versus-rest strategy, comparing each tendon form against all others. Macro-averaged values for precision, recall, the F1 score, and the area under the curve (ROC-AUC) are reported to ensure balanced evaluation across all tendon categories.

To identify misclassification patterns, confusion matrices were generated for each rater and for the majority vote. These matrices were used to visualize common errors and to highlight areas of disagreement or uncertainty between raters.

## Results

### Study cohort

A total of 439 ankle MRI examinations were reviewed. After applying the exclusion criteria, 310 examinations were excluded. We excluded any examination showing signs of peroneal tendon pathology, including abnormal T2 or proton density (PD) signal suggestive of split tear, tenosynovitis, or tendinosis (*n* = 131). Additional exclusion criteria were: fractures (*n* = 35), infections (*n* = 7), ganglion cysts or tumors (*n* = 3), transverse peroneus brevis rupture (*n* = 4), postoperative changes or metal artifacts (*n* = 54), MRI-related artifacts (*n* = 19), multiple MRIs for the same patient (*n* = 33), missing clinical data (*n* = 9), age under 18 years (*n* = 8), and complete rupture of the peroneus longus or brevis tendon (*n* = 6). The final study population comprised 130 examinations performed using our standard ankle protocol for evaluation of trauma or ankle pain (Flowchart in Supplementary material Figure S1). The study cohort consisted of 130 persons with a mean age of 39.7 years and a standard deviation of 13.4 years (range: 18–64 years). There were 73 males (56.2%) and 57 females (43.8%). The left side was examined in 69 persons (53.1%), while the right was examined in 61 persons (46.9%).

### Consensus-based tendon classification

The distribution of tendon forms based on the consensus between raters was as follows: 37.7% of cases (*n* = 49) were classified as general flat, 20.8% (*n* = 27) as flat with a lateral bulge, 19.2% (*n* = 25) as flat with a medial bulge, and 22.3% (*n* = 29) as oval tendon. Figure 1 shows an example of each type.

### Inter-rater agreement

#### Pairwise agreement between raters

Cohen’s kappa—which ranged from 0.589 to 0.865—and Gwet’s AC1—which ranged from 0.580 to 0.865—indicated substantial to almost perfect agreement according to Landis and Koch [27] (Table 1 and Supplementary material Figures S2-S4).

**Table 1:** Agreement statistics for each rater compared with the consensus

Rater*	Cohen’s kappa (*k*)	95% confidence interval	Gwet’s AC1	95% confidence interval
Rater 1	0.823	0.78–0.86	0.823	0.79–0.86
Rater 2	0.793	0.74–0.83	0.791	0.75–0.83
Rater 3	0.707	0.65–0.76	0.705	0.66–0.75
Rater 4	0.741	0.69–0.79	0.739	0.70–0.78
Rater 5	0.781	0.73–0.82	0.781	0.74–0.82
Rater 6	0.865	0.83–0.89	0.865	0.83–0.89
Rater 7	0.589	0.52–0.65	0.580	0.51–0.64

* Raters involved in the classification process: Rater 1—musculoskeletal radiologist with 10 years of experience; Rater 2—physiotherapist; Rater 3—medical doctor; Rater 4—fifth-year medical student; Rater 5—musculoskeletal radiologist with 6 years of experience; Rater 6—radiology resident; Rater 7—physiotherapist

### Intra-rater reliability

After a 3-week washout period, Cohen’s kappa ranged from 0.57 to 0.93, indicating moderate to almost perfect intra-rater agreement. Gwet’s AC1 was generally higher, ranging from 0.70 to 0.93. Most raters demonstrated substantial to almost perfect reproducibility according to Landis and Koch [27].

### Overall agreement across raters

**Fig. 2:**
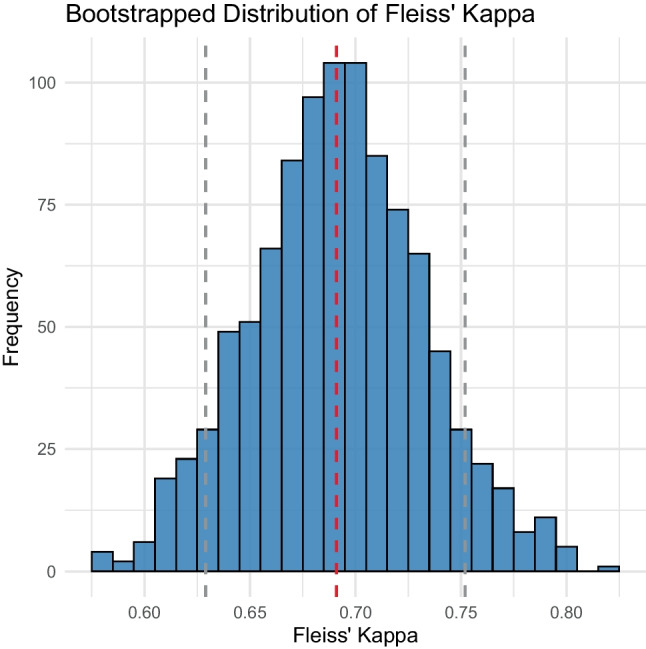
Bootstrapped distribution of Fleiss’ kappa computed from 1000 resamples. The vertical dashed line represents the mean Fleiss’ kappa (0.691), and the grey dashed lines indicate the 95% confidence interval (0.629–0.752)

Fleiss’ kappa across all raters was 0.691 (95% confidence interval 0.629–0.752), indicating substantial agreement according to Landis and Koch [17]. To assess the stability of this result, we performed bootstrapping, resampling the data 1000 times. The 95% confidence interval was 0.629–0.752 (Fig. 2). This narrow confidence interval suggests that the level of agreement between raters is consistent across different resamples. The substantial agreement found, with the lower bound of the confidence interval still above 0.6, indicates reliable rater consistency in classifying the tendon forms.

### Classification performance metrics

The classification performance of individual raters varied, as reflected in the F1 score, harmonic mean, and F2 score. While most raters demonstrated substantial agreement with the consensus classification, we observed differences in balance precision and recall that affected the overall F1 scores (Table 2). Raters 1, 2, and 5 had consistently high performance, with an F1 score > 0.84, indicating strong agreement with the consensus. Rater 6 exhibited the highest classification accuracy, achieving an F1 score of 0.90, suggesting reliable differentiation between tendon forms. Conversely, Raters 3 and 7 had a lower F1 score (0.78 and 0.68, respectively), indicating greater variability in classification, likely due to challenges in distinguishing between certain tendon forms.

**Table 2:** Per-rater and overall performance

Raters*	F1 score	Harmonic mean	F2 score
Rater 1	0.87	0.87	0.87
Rater 2	0.85	0.86	0.84
Rater 3	0.78	0.80	0.78
Rater 4	0.80	0.82	0.80
Rater 5	0.84	0.84	0.84
Rater 6	0.90	0.90	0.90
Rater 7	0.68	0.73	0.68
Overall (majority vote)	0.93	0.93	0.93

* Raters involved in the classification process: Rater 1—musculoskeletal radiologist with 10 years of experience; Rater 2—physiotherapist; Rater 3—medical doctor; Rater 4—fifth-year medical student; Rater 5—musculoskeletal radiologist with 6 years of experience; Rater 6—radiology resident; Rater 7—physiotherapist

To further assess classification reliability, we applied a majority vote approach, using the most frequently assigned class among all raters as the final prediction (Table 2). This method significantly improved classification performance, achieving an F1 score of 0.93, a harmonic mean of 0.935, and an F2 score of 0.929, showing the value of combining multiple assessments to mitigate individual rater variability.

### Classification performance

The ROC curves demonstrated robust classification performance across all tendon forms, with the general flat tendon achieving the steepest curve, indicating clear separability from other classes (Supplementary material Figure S5). A consistently high area under the curve (ROC-AUC = 0.89) confirms that the classification effectively differentiates between tendon forms. It suggests that the classification system provides reliable assessment. The high sensitivity and specificity observed in the ROC analysis highlight the model’s robustness, with a steep slope at a lower false positive rate, indicating minimal misclassification. The cumulative one-versus-rest approach, well suited for these data, ensured that we evaluated classification performance in a clinically meaningful way rather than treating each category as an independent class.

The classification system demonstrated strong overall performance, with an accuracy of 0.83 and a macro-averaged F1 score of 0.83, indicating well-balanced performance across the tendon categories. The macro-averaged precision and recall were 0.84 and 0.83, respectively. The area under the receiver operating characteristic curve was 0.89. A null accuracy of 0.31 confirmed that the classification system substantially outperformed random chance.

### Precision-recall analysis

We used a precision-recall curve to evaluate classification performance; it demonstrates the trade-off between recall and precision. The analysis revealed differences in precision, recall, and the F1 score across the tendon forms. We observed higher F1 scores (Supplementary material Figure S6) for tendon forms with more distinct shapes, whereas lower F1 scores reflected greater uncertainty in classification. The high area under the precision-recall curve (0.83) indicates good performance in correctly identifying tendon forms while reducing false positives.

### Error analysis and misclassification trends

**Fig. 3:**
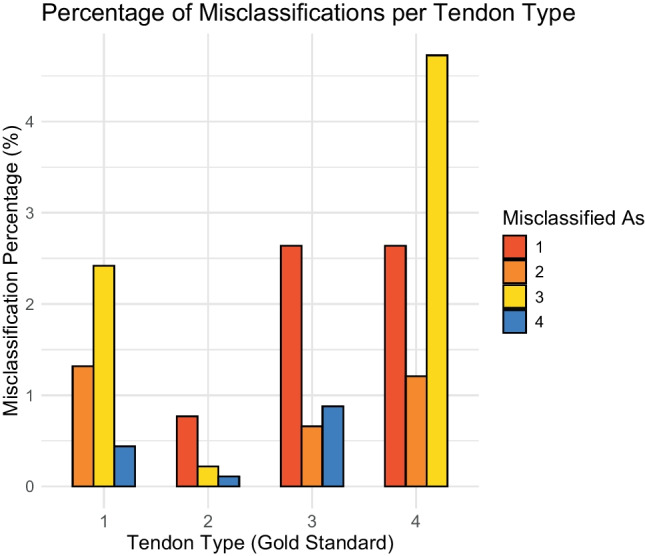
Bar chart showing the percentage (%) of times each tendon form was classified incorrectly by the raters. Tendon forms: 1, general flat; 2, flat with a lateral bulge; 3, flat with a medial bulge; and 4, oval tendon

We generated a confusion matrix for individual raters (Supplementary material Figure S7) to complement Fleiss’ kappa and to provide a more detailed analysis of the misclassifications. The most common error—misclassifying the oval tendon as general flat with a medial bulge (4.76%)—occurred almost twice as much as the other errors (Fig. 3). The confusion matrix shows the frequency with which raters classified tendons as another type, indicating both areas of agreement and misclassification. The diagonal of the matrix, representing the correctly classified tendon forms, was consistently high, suggesting that raters generally agreed with the gold standard (the consensus between two musculoskeletal radiologists).

## Discussion

The most important finding of this study is that the proposed MRI-based classification system for peroneus brevis tendon morphology is reliable and reproducible, with substantial inter-rater agreement. MRI plays a central role in sports imaging, offering detailed assessment of soft tissue structures such as tendons. Previous studies, including that by Ersoz et al., have used descriptive terms such as “boomerang-shaped” to characterize peroneus brevis split tear on axial MRI [28]. While these observations suggest an association with split tear, a standardized MRI classification system has been lacking. Our study addresses this gap by proposing and validating a reproducible four-type classification based on cross-sectional tendon shape, providing a consistent background for both clinical assessment and future research.

The substantial agreement based on Fleiss’ kappa suggests that, overall, the raters were able to reliably classify the tendon forms according to the consensus. The consistently higher Gwet’s AC1 values may highlight the limitations of Cohen’s Kappa in cases with uneven category distributions [29]. These findings suggest that individual raters maintained consistency in their assessments, reinforcing the reliability of the classification system. Minor discrepancies may be related to borderline tendon forms or visual assessment, warranting future metric standardization efforts to further optimize classification consistency. To better understand which types were most frequently confused with one another, we constructed a confusion matrix.

The inclusion of non-radiologists among the raters was intended to assess the intuitiveness and reproducibility of the classification system across varying levels of training and clinical backgrounds. Although our rater group did not include any orthopedic surgeons, it did include two physiotherapists, offering a balanced mix of raters. Had we included only experienced radiologists, inter-rater agreement might have been higher; however, the findings would have been less generalizable to everyday clinical practice in sports medicine, where patients are managed by multidisciplinary teams and MRI is interpreted by different healthcare professionals [30].

Some previous studies have referred to the different forms of the peroneus brevis tendon on transverse cross section, but no validated classification exists [31]. Overly complex descriptions of tendon shape can lead to misunderstandings. Therefore, it is necessary to develop a relatively simple classification that still captures the complexity of anatomical variation. To our knowledge, this is the first validated classification of peroneus brevis tendon morphology at the lateral malleolus. We choose this level based on clinical observation and the fact that this is the most common location of a peroneus brevis split tear [32, 33].

While the overall agreement was substantial, we observed variability among the raters, reflecting the challenges of distinguishing subtle anatomical differences [27]. The misclassification patterns seen in the confusion matrix show the limitations of the visual assessment [34]. The confusion matrix analysis revealed that the oval tendon and the general flat tendon with a medial bulge were frequently misclassified, indicating some similarity in visual classification. This overlap contributed to lower inter-rater agreement and highlights the diagnostic challenges faced in sports medicine when evaluating subtle tendon pathology.

Despite anatomical variations, our analysis demonstrated that the classification remains reliable even when radiologists represent a minority within the group of raters. Multicenter studies with larger and more diverse samples would help confirm whether these misclassification patterns are generalizable across different patient populations. Nevertheless, we determined the sample size prior to commencing the study to ensure we had adequate statistical power and that our results are valid.

Our raters did not perform tendon measurements, as we deliberately chose a purely visual assessment method to reflect real-world clinical scenarios where decisions must be time effective. We hypothesize that, in clinical practice, most raters rely on visual evaluation, occasionally supported by measurements. It is possible that using measurements could have further improved agreement. We think that in the future, the use of artificial intelligence may be used to classify tendon types. However, at present, no such solution exists.

The bootstrapping analysis also highlighted the stability of Fleiss’ kappa, confirming the robustness of our classification system [35]. The narrow confidence intervals and similar Cohen’s kappa and Gwet’s AC1 indicate that the observed level of agreement is unlikely to be due to sampling variability and can be generalized beyond our study cohort. The strong correlation between these two agreement measures confirms the consistency and robustness of the proposed classification system, supporting its potential for additional studies and clinical implementation.

The overlap in the ROC curves suggests some degree of uncertainty in classification between similar tendon forms, which we believe could be further refined with measurements or machine learning models.

Despite the strengths of this study, several limitations should be acknowledged. First, the classification is based solely on axial MRI without surgical correlation. However, because this study focused on tendons without imaging evidence of pathology, surgical exploration would not be ethically appropriate. Second, the classification relied on a single, pre-selected axial slice. While this may not capture the full morphological variability in pathological cases, our preliminary analysis demonstrated that the cross-sectional shape of the peroneus brevis tendon remains consistent within the retromalleolar segment in normal tendons. Third, the classification was based on visual assessment, which introduces a degree of subjectivity. Although inter- and intra-rater agreement was strong, future studies may benefit from incorporating quantitative morphometric analysis to enhance reproducibility. Finally, the included MRIs were obtained for clinical indications, which may introduce selection bias. Further prospective validation using standardized imaging protocols and inclusion of pathological cases is warranted to refine and expand the classification system.

The motivation to develop this classification system arose from our clinical observation that peroneus brevis split tears frequently appear in tendons with a flattened morphology. Although the current study was designed to assess the reproducibility of the classification in a non-pathological cohort, we have also performed a separate, preliminary analysis in a matched group of patients with and without split tears. These early findings indicate that a flat-shaped tendon is more frequently observed in cases with peroneus brevis split tears on MRI. Although preliminary, this observation underscores the potential clinical relevance of the classification and supports its further evaluation as a prognostic imaging marker.

Our classification system for the peroneus brevis tendon proved to be a robust and reliable tool, demonstrating substantial inter-rater agreement and excellent overall performance. Misclassifications were rare and mostly limited to tendon types with closely related anatomical features. Its simplicity, practicality, and reproducibility make it well-suited for both clinical and research use. The system’s strength lies in its ability to capture complex anatomical variation in a clear and accessible format. With appropriate rater training, it offers consistent and accurate tendon evaluation. Future studies should investigate the performance of this classification system across other imaging modalities, including ultrasound, and in diverse international settings.

## Supplementary Information

Below is the link to the electronic supplementary material.

ESM1 Fig. S1. Flowchart (PDF 22.6 KB)

ESM2 Fig. S2. Comparison of Cohen’s kappa and Gwet’s AC1 for each rater versus the consensus. Raters: Rater 1 – musculoskeletal radiologist with 10 years of experience; Rater 2 – physiotherapist; Rater 3 – medical doctor; Rater 4 – fifth-year medical student; Rater 5 – musculoskeletal radiologist with 6 years of experience; Rater 6 – radiology resident; Rater 7 – physiotherapist (PDF 23.2 KB)

ESM3 Fig. S3. Heatmap visualisation of pairwise Cohen’s kappa between raters. Darker colours indicate stronger agreement. Raters: Rater 1 – musculoskeletal radiologist with 10 years of experience; Rater 2 – physiotherapist; Rater 3 – medical doctor; Rater 4 – fifth-year medical student; Rater 5 – musculoskeletal radiologist with 6 years of experience; Rater 6 – radiology resident; Rater 7 – physiotherapist (PDF 30.2 KB)

ESM4 Fig. S4. Heatmap visualisation of pairwise Gwet’s AC1 between raters. Darker colours indicate stronger agreement. Raters: Rater 1 – musculoskeletal radiologist with 10 years of experience; Rater 2 – physiotherapist; Rater 3 – medical doctor; Rater 4 – fifth-year medical student; Rater 5 – musculoskeletal radiologist with 6 years of experience; Rater 6 – radiology resident; Rater 7 – physiotherapist (PDF 29.5 KB)

ESM5 Fig. S5. Receiver operating characteristic (ROC) curve for the tendon classification system, using a cumulative one-versus-rest approach. Tendon forms: 1, general flat; 2, flat with a lateral bulge; 3, flat with a medial bulge; and 4, oval tendon. Sensitivity (the true positive rate) is plotted against 1 – specificity (the false positive rate). The curve demonstrates the ability of the classification model to distinguish between the four tendon forms (PDF 19.4 KB)

ESM6 Fig. S6. The average precision-recall curve demonstrating the relationship between recall and precision for tendon classification for all tendon forms (average curve). The curve shows the trade-off between recall and precision at different classification thresholds, with higher values indicating better performance in distinguishing tendon forms. AUC, area under the curve (PDF 28.2 KB)

ESM7 Fig. S7. Confusion matrix heatmap illustrating classification discrepancies between each rater, the overall evaluation (majority vote), and the consensus classification. Tendon forms: 1, general flat; 2, flat with a lateral bulge; 3, flat with a medial bulge; and 4, oval tendon. Darker colours indicate higher agreement with the consensus, while lighter shades highlight misclassifications. Raters involved in the classification process: Rater 1 – musculoskeletal radiologist with 10 years of experience; Rater 2 – physiotherapist; Rater 3 – medical doctor; Rater 4 – fifth-year medical student; Rater 5 – musculoskeletal radiologist with 6 years of experience; Rater 6 – radiology resident; Rater 7 – physiotherapist (PDF 41.7 KB)

## Data Availability

The data supporting this case report are available from the corresponding author on reasonable request, in compliance with the Ethics Committee’s guidelines to ensure patient confidentiality.
