# Supplementary figures and images for: Validation of an MRI-based classification of peroneus brevis tendon morphology: a four-type system with high inter-rater reliability
Source: Skeletal Radiol. 2025 Aug 13;55(1):205–14. doi: 10.1007/s00256-025-05010-4 (PMC12627172; doi:10.1007/s00256-025-05010-4)

# Flowchart in the study

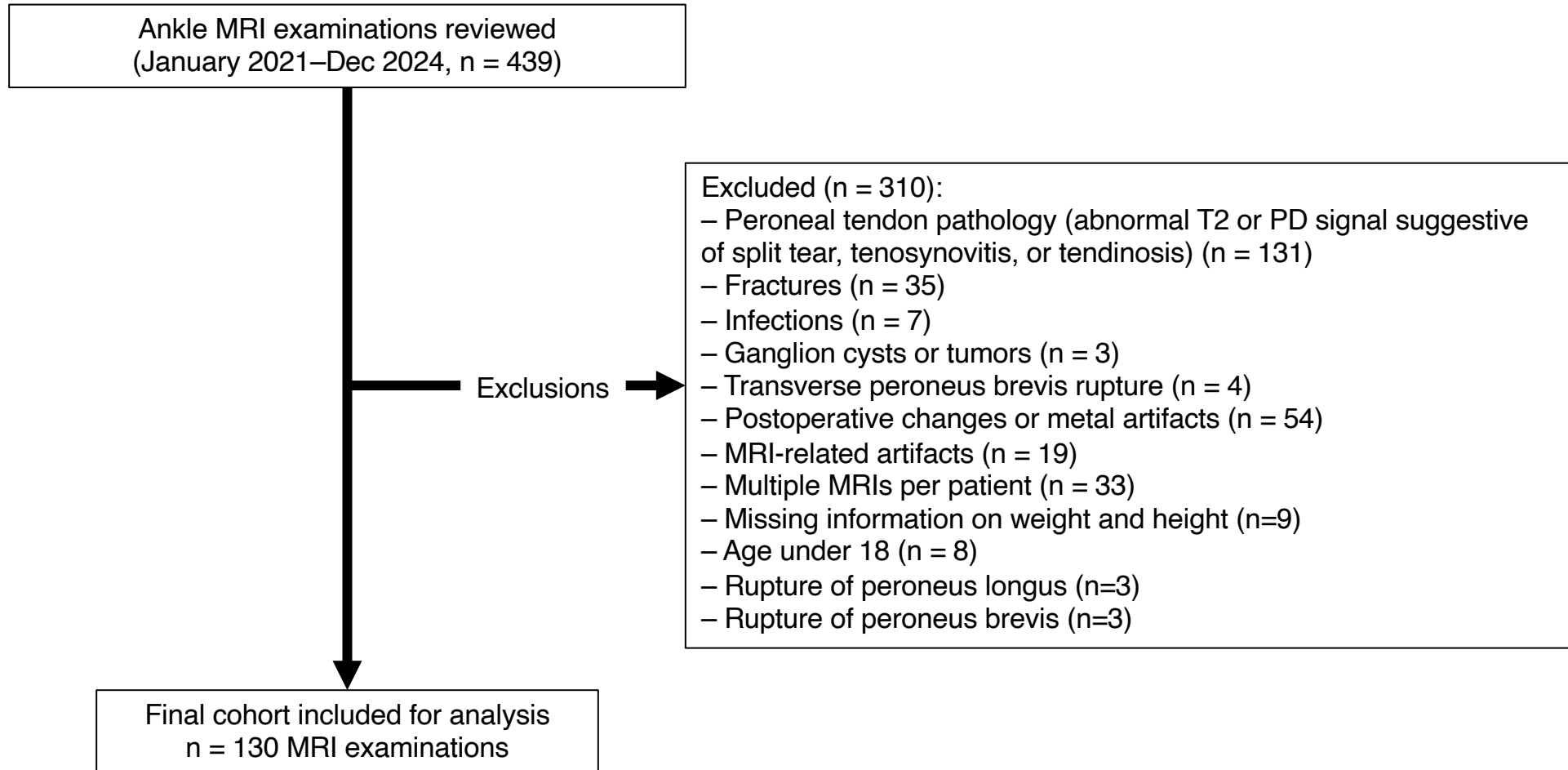

Supplement: Supplementary file 1 — Fig. S1. Flowchart (PDF 22.6 KB) [file 256_2025_5010_MOESM1_ESM.pdf]

Cohen's Kappa & Gwet's AC1 for Each Rater vs. Consensus

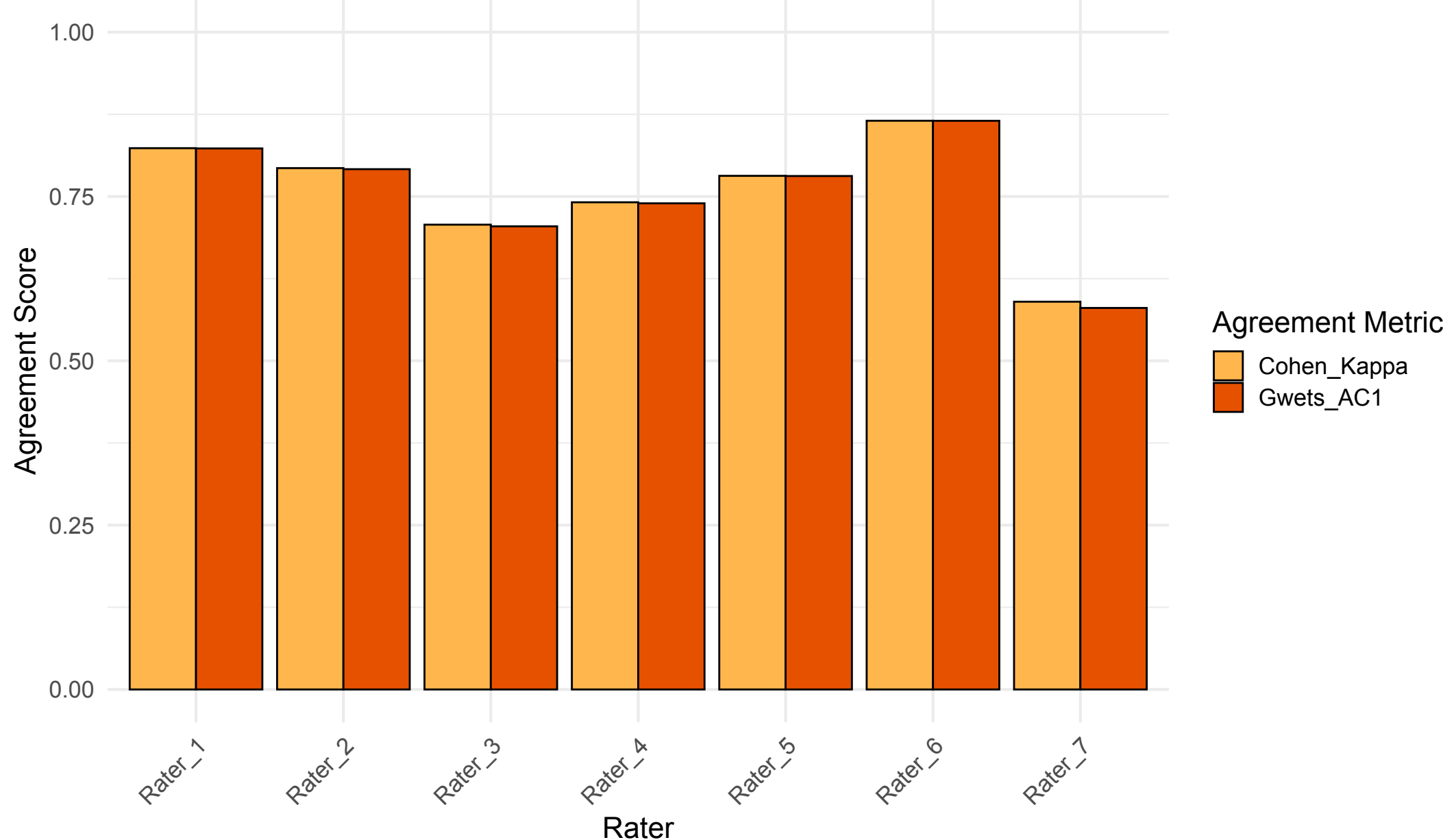

Supplement: Supplementary file 2 — Fig. S2. Comparison of Cohen’s kappa and Gwet’s AC1 for each rater versus the consensus. Raters: Rater 1 – musculoskeletal radiologist with 10 years of experience; Rater 2 – physiotherapist; Rater 3 – medical doctor; Rater 4 – fifth-year medical student; Rater 5 – musculoskeletal radiologist with 6 years of experience; Rater 6 – radiology resident; Rater 7 – physiotherapist (PDF 23.2 KB) [file 256_2025_5010_MOESM2_ESM.pdf]

Cohen's Kappa Heatmap Between Raters

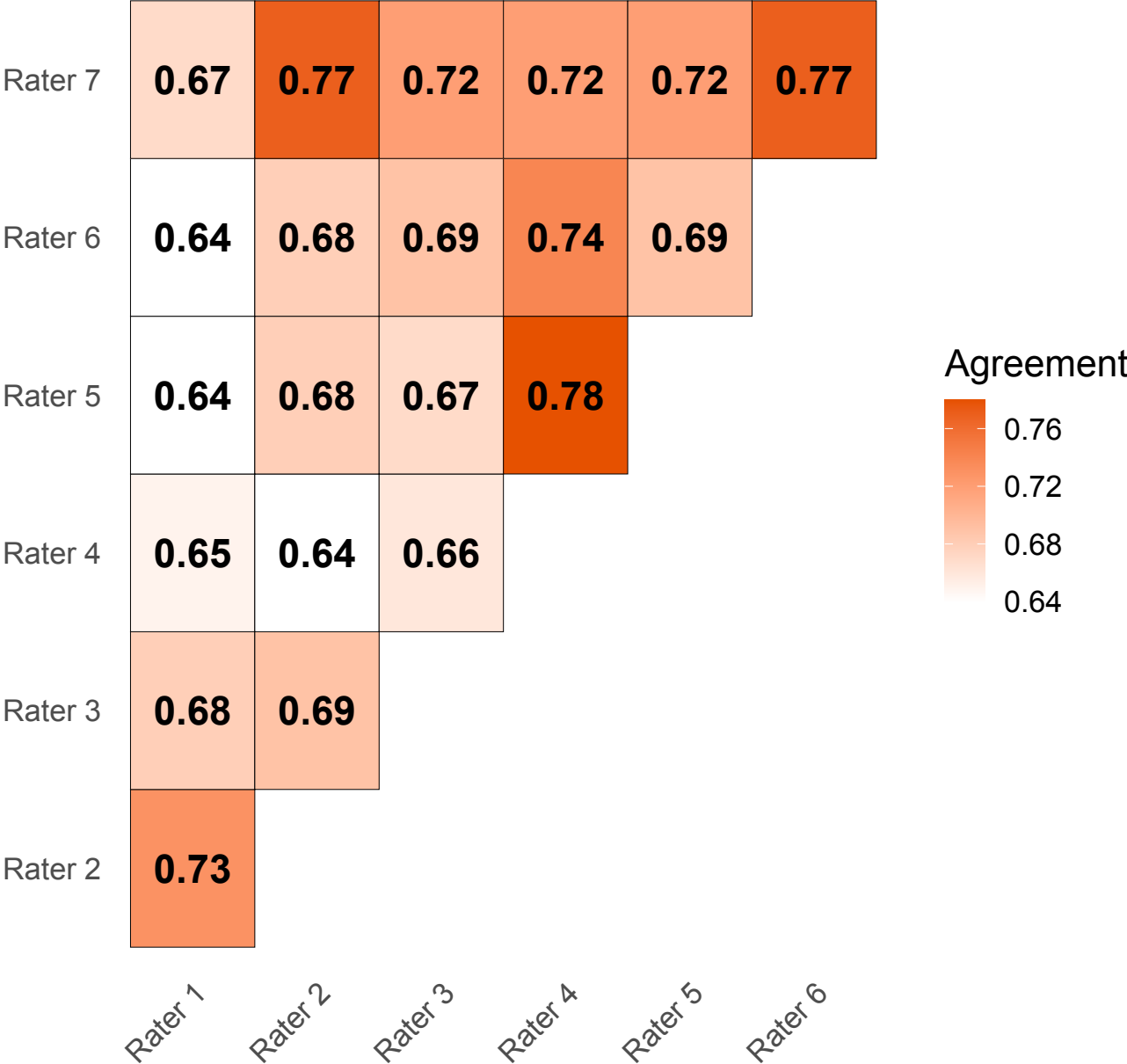

Supplement: Supplementary file 3 — Fig. S3. Heatmap visualisation of pairwise Cohen’s kappa between raters. Darker colours indicate stronger agreement. Raters: Rater 1 – musculoskeletal radiologist with 10 years of experience; Rater 2 – physiotherapist; Rater 3 – medical doctor; Rater 4 – fifth-year medical student; Rater 5 – musculoskeletal radiologist with 6 years of experience; Rater 6 – radiology resident; Rater 7 – physiotherapist (PDF 30.2 KB) [file 256_2025_5010_MOESM3_ESM.pdf]

Gwet's AC1 Heatmap Between Raters

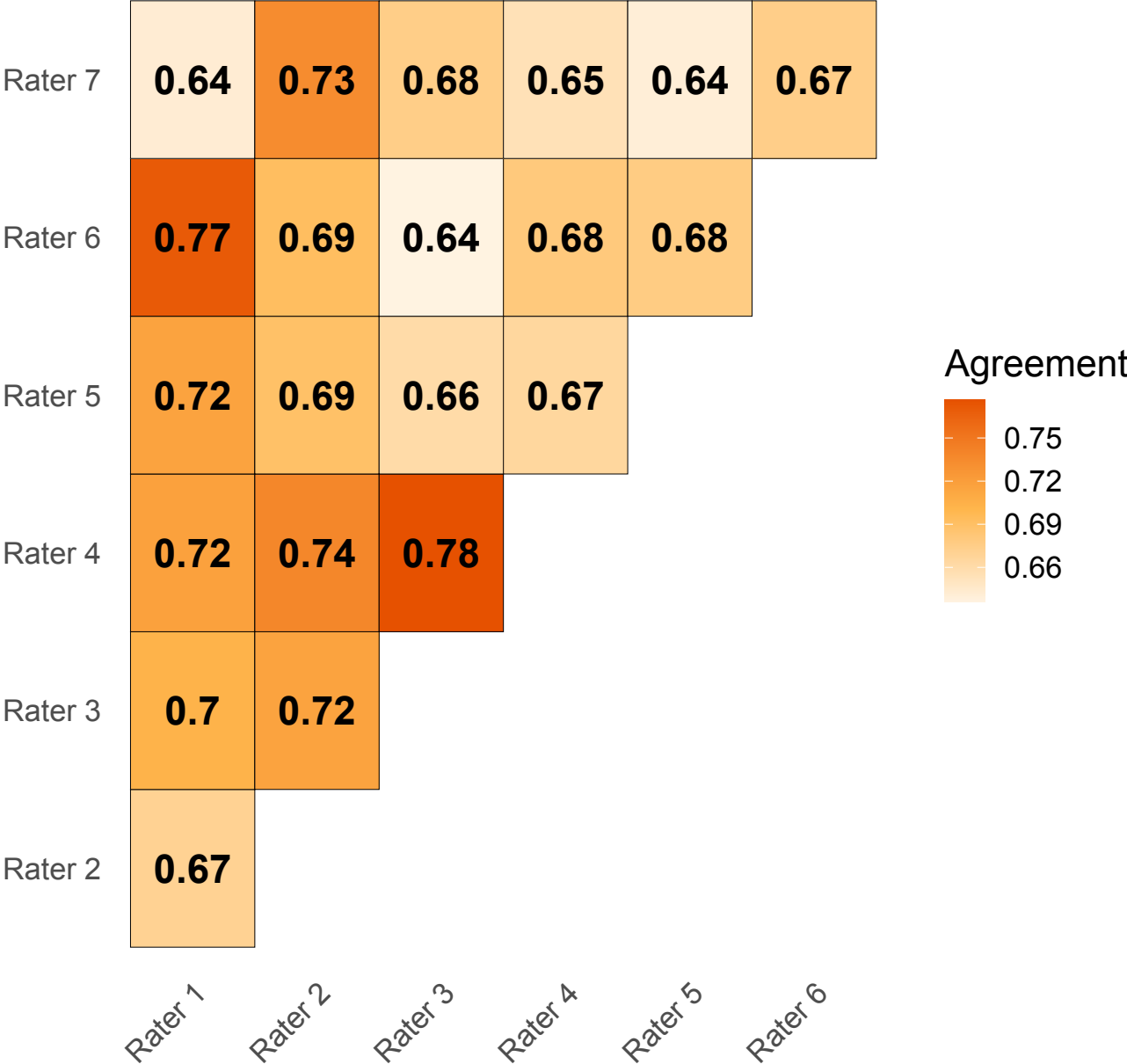

Supplement: Supplementary file 4 — Fig. S4. Heatmap visualisation of pairwise Gwet’s AC1 between raters. Darker colours indicate stronger agreement. Raters: Rater 1 – musculoskeletal radiologist with 10 years of experience; Rater 2 – physiotherapist; Rater 3 – medical doctor; Rater 4 – fifth-year medical student; Rater 5 – musculoskeletal radiologist with 6 years of experience; Rater 6 – radiology resident; Rater 7 – physiotherapist (PDF 29.5 KB) [file 256_2025_5010_MOESM4_ESM.pdf]

Ordinal ROC Curve (Cumulative One-vs-Rest)

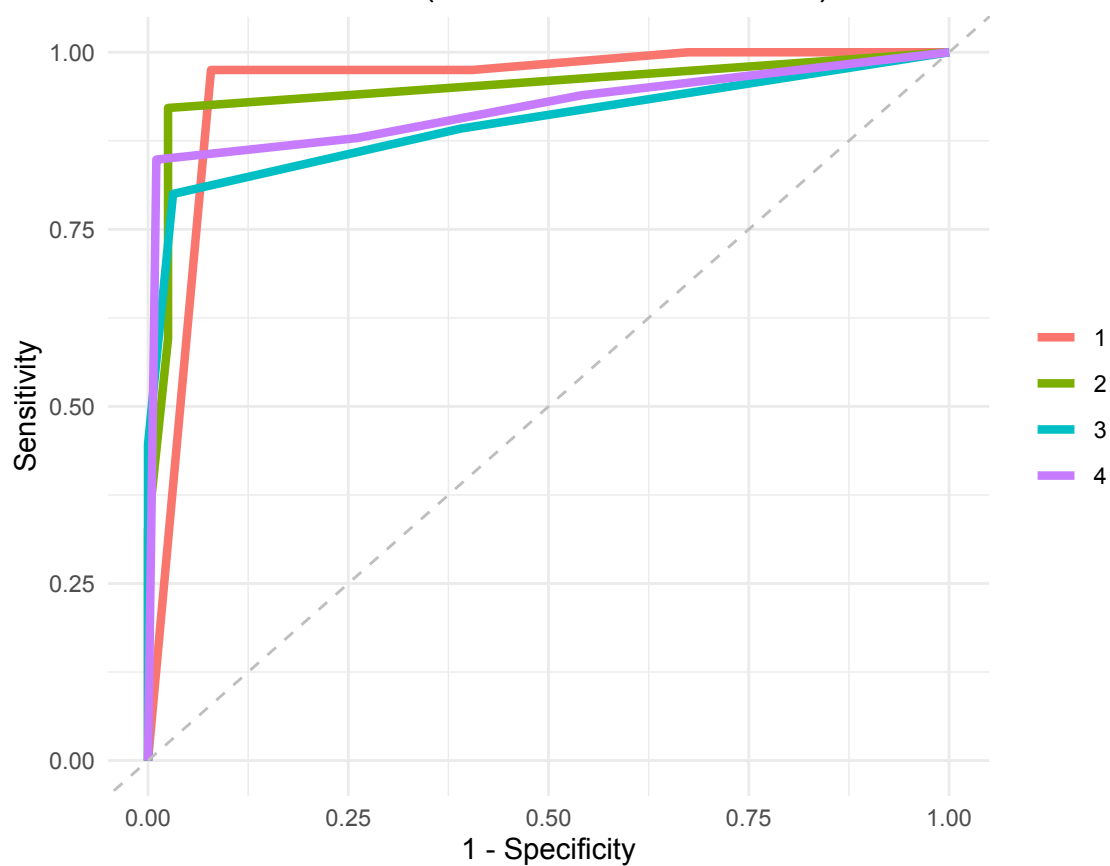

Supplement: Supplementary file 5 — Fig. S5. Receiver operating characteristic (ROC) curve for the tendon classification system, using a cumulative one-versus-rest approach. Tendon forms: 1, general flat; 2, flat with a lateral bulge; 3, flat with a medial bulge; and 4, oval tendon. Sensitivity (the true positive rate) is plotted against 1 – specificity (the false positive rate). The curve demonstrates the ability of the classification model to distinguish between the four tendon forms (PDF 19.4 KB) [file 256_2025_5010_MOESM5_ESM.pdf]

# Precision-Recall Curve

AUC = 0.8284904

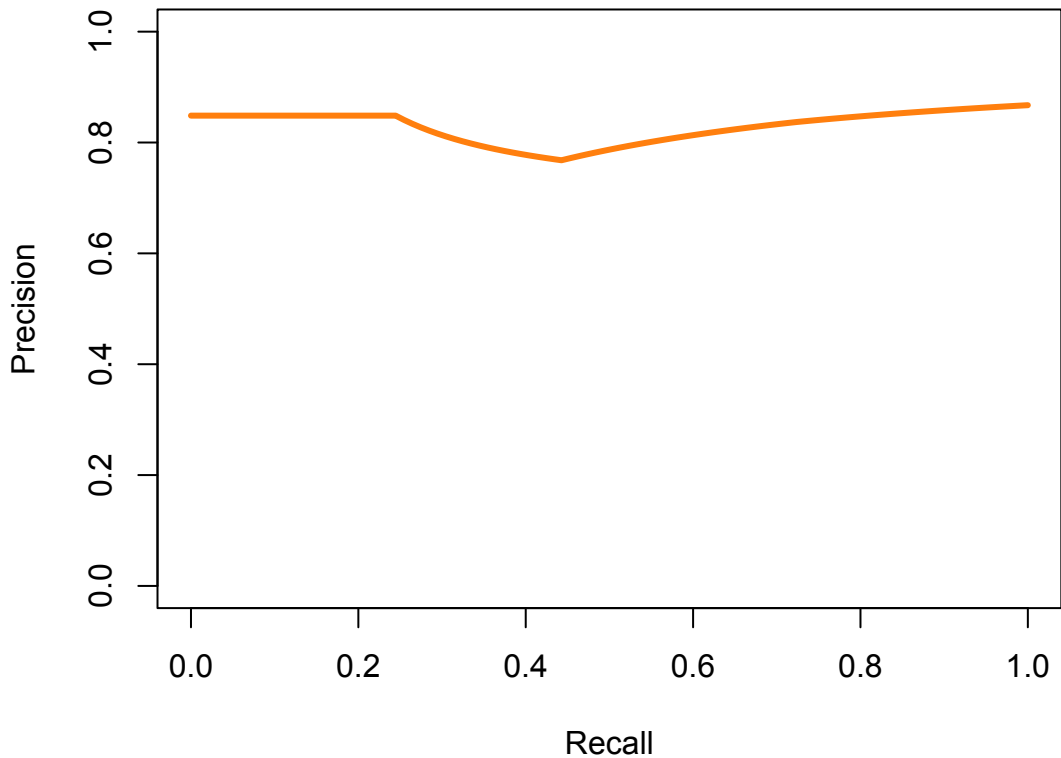

Supplement: Supplementary file 6 — Fig. S6. The average precision-recall curve demonstrating the relationship between recall and precision for tendon classification for all tendon forms (average curve). The curve shows the trade-off between recall and precision at different classification thresholds, with higher values indicating better performance in distinguishing tendon forms. AUC, area under the curve (PDF 28.2 KB) [file 256_2025_5010_MOESM6_ESM.pdf]
