# Supplementary material for: Validation of an MRI-based classification of peroneus brevis tendon morphology: a four-type system with high inter-rater reliability
Source: Skeletal Radiol. 2025 Aug 13;55(1):205–14. doi: 10.1007/s00256-025-05010-4 (PMC12627172; doi:10.1007/s00256-025-05010-4)
Supplement: Supplementary file 7 — Fig. S7. Confusion matrix heatmap illustrating classification discrepancies between each rater, the overall evaluation (majority vote), and the consensus classification. Tendon forms: 1, general flat; 2, flat with a lateral bulge; 3, flat with a medial bulge; and 4, oval tendon. Darker colours indicate higher agreement with the consensus, while lighter shades highlight misclassifications. Raters involved in the classification process: Rater 1 – musculoskeletal radiologist with 10 years of experience; Rater 2 – physiotherapist; Rater 3 – medical doctor; Rater 4 – fifth-year medical student; Rater 5 – musculoskeletal radiologist with 6 years of experience; Rater 6 – radiology resident; Rater 7 – physiotherapist (PDF 41.7 KB) [file 256_2025_5010_MOESM7_ESM.pdf]

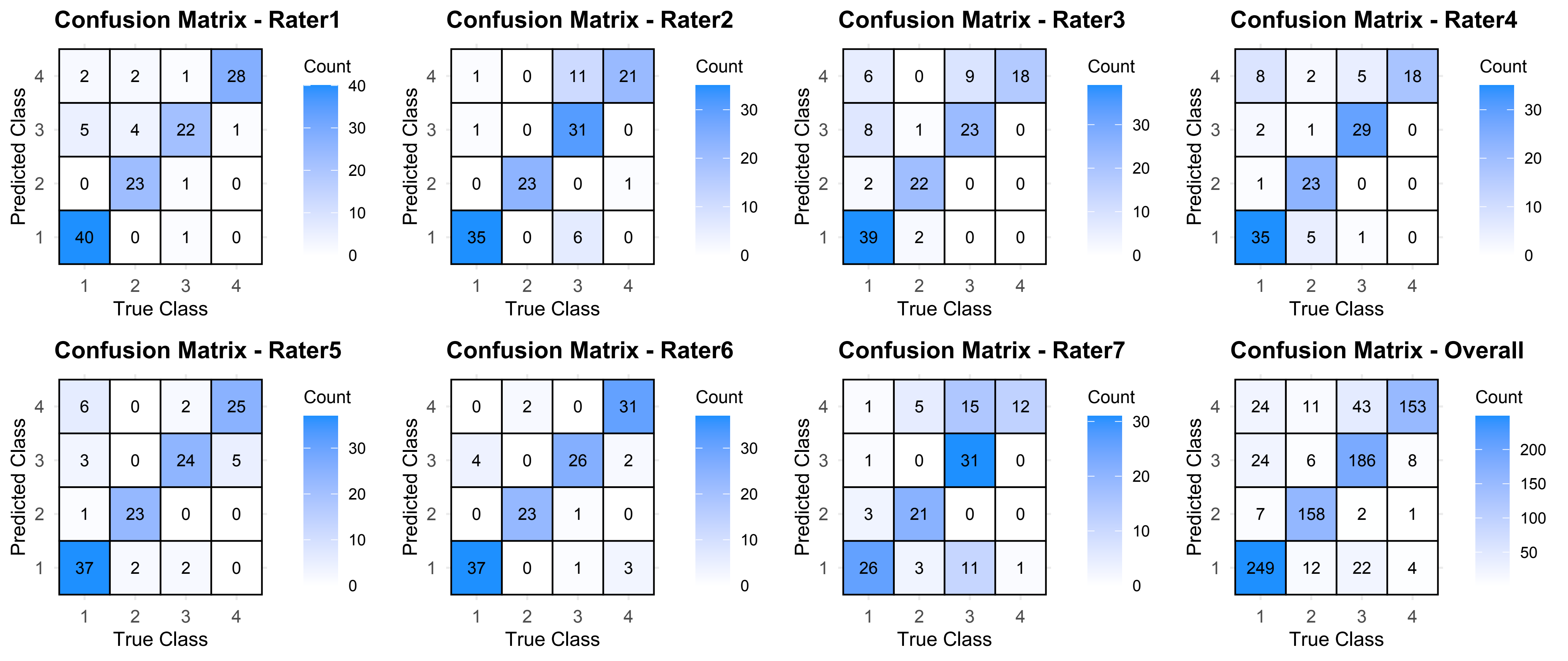
Confusion Matrix - Rater1
Predicted Class 4: 2 2 1 28
Predicted Class 3: 5 4 22 1
Predicted Class 2: 0 23 1 0
Predicted Class 1: 40 0 1 0
True Class 1 2 3 4
Count 0 10 20 30 40
Confusion Matrix - Rater2
Predicted Class 4: 1 0 11 21
Predicted Class 3: 1 0 31 0
Predicted Class 2: 0 23 0 1
Predicted Class 1: 35 0 6 0
True Class 1 2 3 4
Count 0 10 20 30
Confusion Matrix - Rater3
Predicted Class 4: 6 0 9 18
Predicted Class 3: 8 1 23 0
Predicted Class 2: 2 22 0 0
Predicted Class 1: 39 2 0 0
True Class 1 2 3 4
Count 0 10 20 30
Confusion Matrix - Rater4
Predicted Class 4: 8 2 5 18
Predicted Class 3: 2 1 29 0
Predicted Class 2: 1 23 0 0
Predicted Class 1: 35 5 1 0
True Class 1 2 3 4
Count 0 10 20 30
Confusion Matrix - Rater5
Predicted Class 4: 6 0 2 25
Predicted Class 3: 3 0 24 5
Predicted Class 2: 1 23 0 0
Predicted Class 1: 37 2 2 0
True Class 1 2 3 4
Count 0 10 20 30
Confusion Matrix - Rater6
Predicted Class 4: 0 2 0 31
Predicted Class 3: 4 0 26 2
Predicted Class 2: 0 23 1 0
Predicted Class 1: 37 0 1 3
True Class 1 2 3 4
Count 0 10 20 30
Confusion Matrix - Rater7
Predicted Class 4: 1 5 15 12
Predicted Class 3: 1 0 31 0
Predicted Class 2: 3 21 0 0
Predicted Class 1: 26 3 11 1
True Class 1 2 3 4
Count 0 10 20 30
Confusion Matrix - Overall
Predicted Class 4: 24 11 43 153
Predicted Class 3: 24 6 186 8
Predicted Class 2: 7 158 2 1
Predicted Class 1: 249 12 22 4
True Class 1 2 3 4
Count 50 100 150 200
